# Terminal Phenoxy Group as a Privileged Moiety of the Drug Scaffold—A Short Review of Most Recent Studies 2013–2022

**DOI:** 10.3390/ijms23168874

**Published:** 2022-08-09

**Authors:** Paweł Kozyra, Monika Pitucha

**Affiliations:** Independent Radiopharmacy Unit, Faculty of Pharmacy, Medical University of Lublin, 20-093 Lublin, Poland

**Keywords:** phenoxy group, drug scaffold, anticancer activity, neurological disorder, anti-HIV activity, antimicrobial, analgesic activity, lymphoma, antidiabetic, adrenergic receptor activity

## Abstract

The terminal phenoxy group is a moiety of many drugs in use today. Numerous literature reports indicated its crucial importance for biological activity; thus, it is a privileged scaffold in medicinal chemistry. This review focuses on the latest achievements in the field of novel potential agents bearing a terminal phenoxy group in 2013–2022. The article provided information on neurological, anticancer, potential lymphoma agent, anti-HIV, antimicrobial, antiparasitic, analgesic, anti-diabetic as well as larvicidal, cholesterol esterase inhibitors, and antithrombotic or agonistic activities towards the adrenergic receptor. Additionally, for selected agents, the Structure–Activity–Relationship (SAR) is also discussed. Thus, this study may help the readers to better understand the nature of the phenoxy group, which will translate into rational drug design and the development of a more efficient drug. To the best of our knowledge, this is the first review devoted to an in-depth analysis of the various activities of compounds bearing terminal phenoxy moiety.

## 1. Introduction

In 2021, the Food and Drug Administration (FDA) approved 50 new drugs [1]. Based on the Biotechnology Innovation Organization study in 2006–2015, only 9.6% of drugs have successfully completed clinical trials. Insufficient effectiveness in the treatment of a given disease is responsible for 30% of drug failures [2]. Harrison’s analysis in 2013–2015 indicated that the failure rate between phase 2 and phase 3 of clinical trials concerned more than half of drug candidates [3]. The other side of the coin are the factors that render current medications ineffective. The antibiotic crisis related to the growing resistance of bacteria is becoming common [4] and once again bacterial infections are lethal [5]. The situation is similar with oncological drugs. Related to the uniqueness of each tumor, we observe as many resistance mechanisms as there are cancer patients [6]—not to mention emerging new diseases or even pandemics as is currently in the case with SARS-CoV-2. For two years, it has claimed about 6.3 million victims [7]. This indicated the urgent need to search for new, more effective therapeutic agents. Novel drugs should be a response to the resistance and ineffectiveness of those currently used.

**Figure 1:**
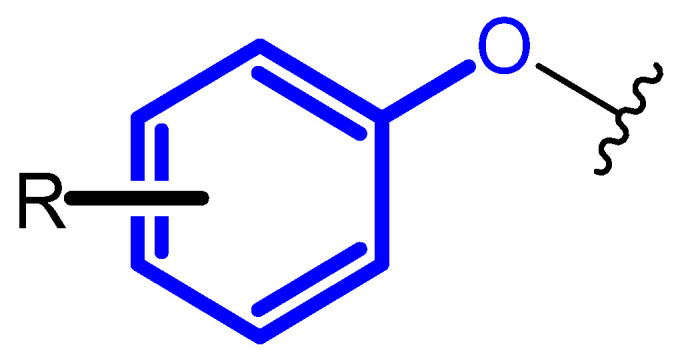
Unsubstituted terminal phenoxy group or substituted with R substituent.

One method of optimizing the lead structure is to introduce new substituents, which resulted in different shape, conformation, and created interaction with the drug target [8,9]. It may also provide significant changes in the pharmacokinetic profile of the drug [10]. The introduction of terminal phenoxy group into the drug scaffold may respond to these needs. Phenoxy moiety is presented in Figure 1.

The phenoxy group is a key pharmacophore component for many drugs that are used today, such as viral drugs [11,12], prostate relievers [13], or Bruton kinase inhibitors [14]. Moreover, compounds bearing this group possessed anti-inflammatory properties [15]. There are more and more papers demonstrating the key nature of the phenoxy moiety for the biological activity of the compound. Most often, the presence of the phenoxy moiety provided the chances for the compound to match the target, ensuring selectivity, the π–π interaction, or increase the ability to form the hydrogen bonds by the oxygen ether atom. The future of pharmacotherapy may be a new class of drugs bearing the terminal phenoxy group.

In our review, we focused on the latest achievements in the field of novel potential agents bearing a terminal phenoxy group in 2013–2022. The first part is a compilation of the currently FDA-approved drugs bearing the terminal phenoxy group, the second part concerns the ingredients of cosmetics, and, in the final part, the most promising compounds from most recent studies are presented.

## 2. Compound Bearing Terminal Phenoxy Group Currently in Use

### 2.1. FDA-Approved Drugs

**Figure 2:**
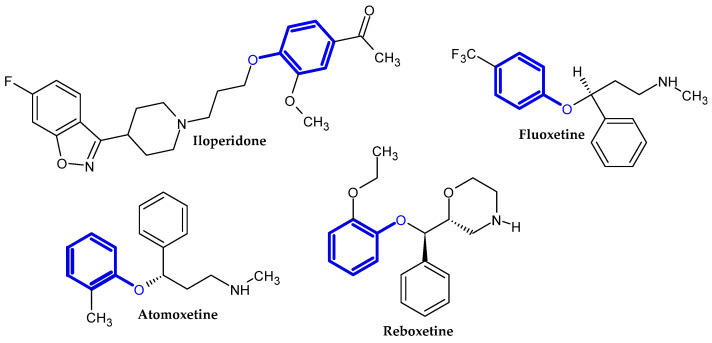
Drugs currently used in the treatment of neurological disorders.

**Figure 3:**
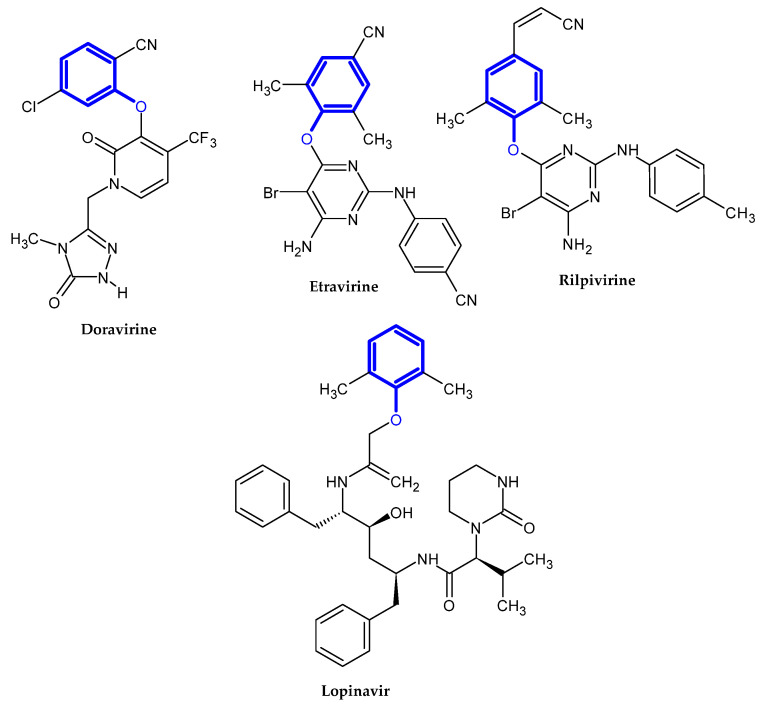
Antiviral drugs.

**Figure 4:**
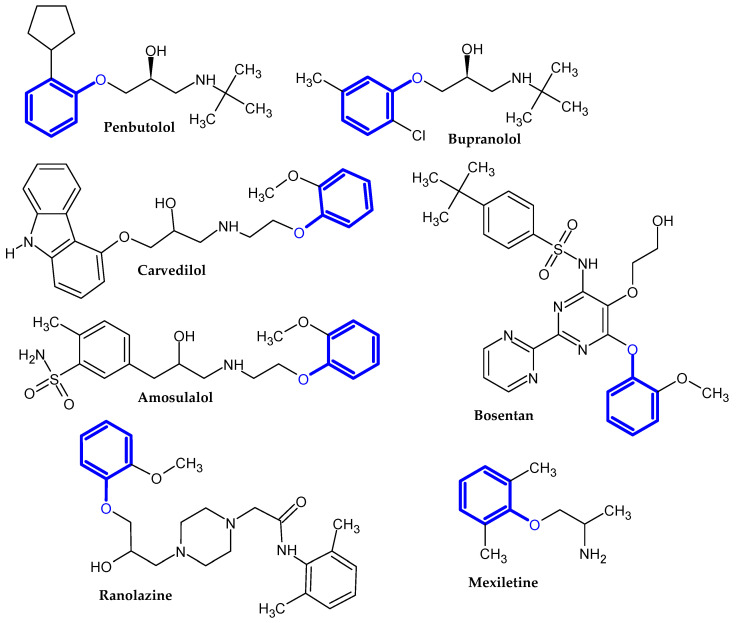
Cardiac drugs.

**Figure 5:**
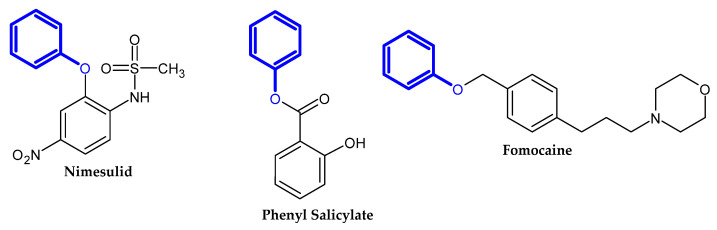
Analgesic drugs currently used in treatment.

**Figure 6:**
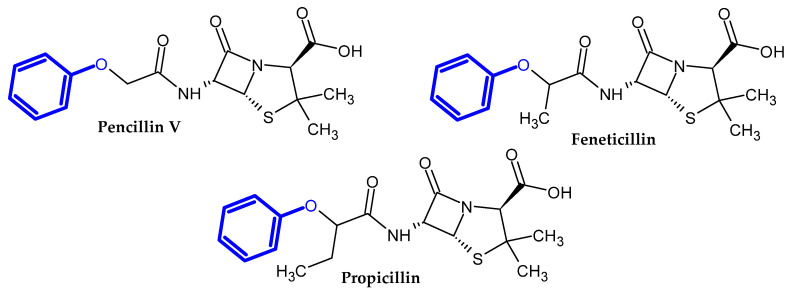
Antimicrobial drugs.

**Figure 7:**
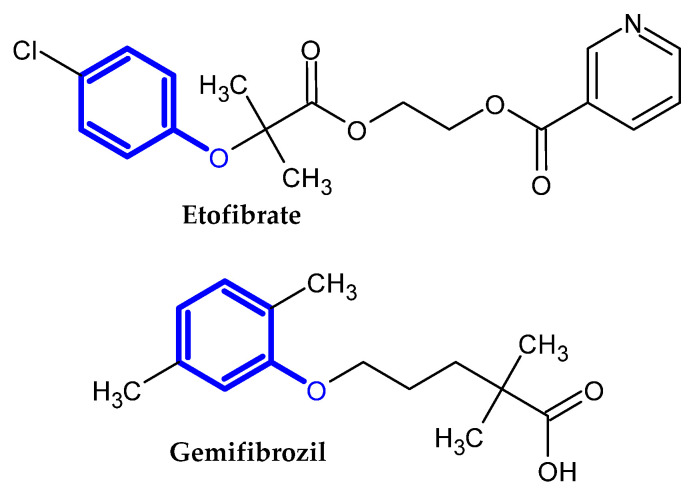
Anti-cholesterol drugs.

**Figure 8:**
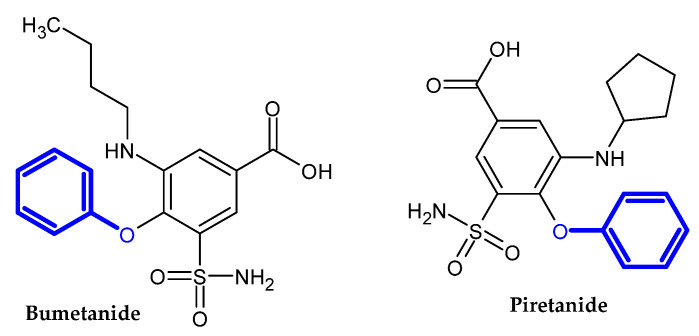
Diuretic drugs.

**Figure 9:**
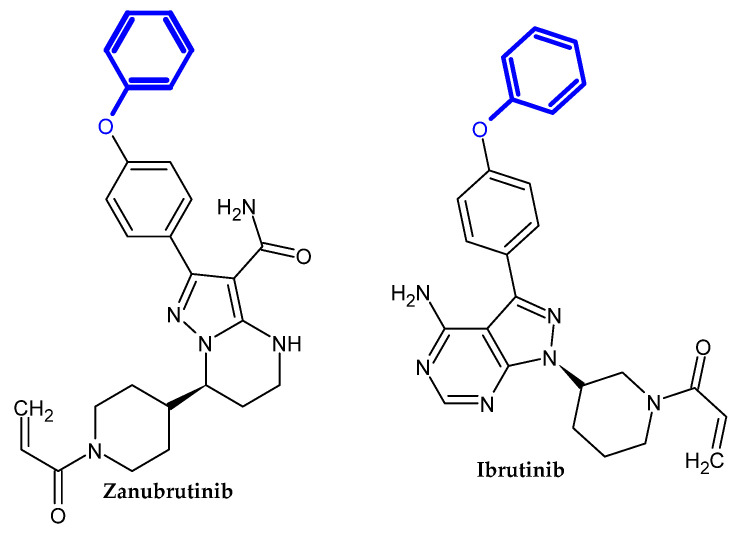
Anti-leukemia drugs.

**Figure 10:**
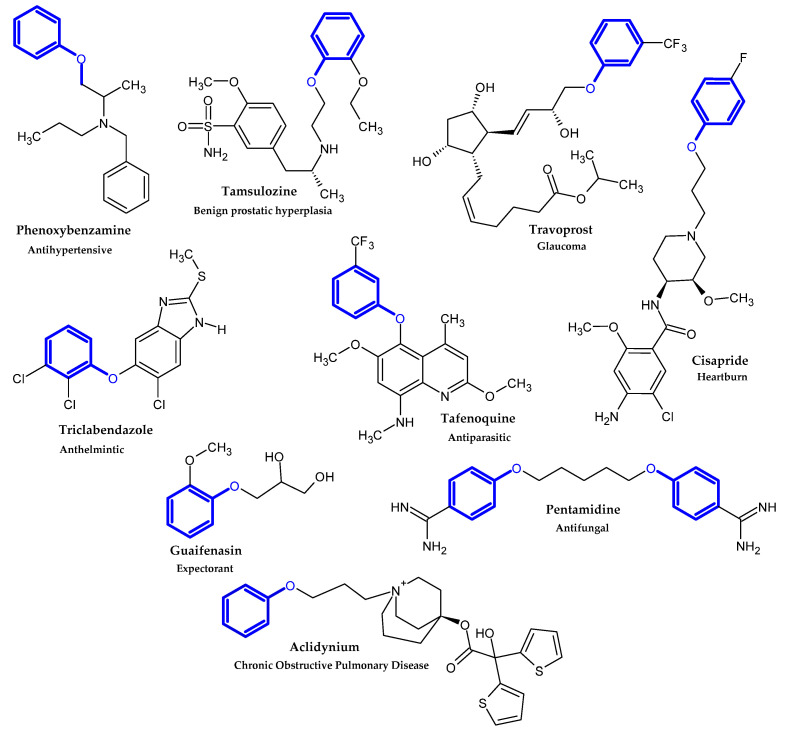
Other drugs with various biological activities.

In the previous section, we mentioned some classes of drugs bearing the phenoxy group. Now, we present a comprehensive list of drugs currently used in treatment, broken down into groups by biological activity. Drugs for treatment neurological disorder, antiviral, cardiac, analgesic, antimicrobial, anti-cholesterol, diuretic, anti-leukemia, and other drugs with various biological activities are presented in Figure 2, Figure 3, Figure 4, Figure 5, Figure 6, Figure 7, Figure 8, Figure 9 and Figure 10, respectively.

### 2.2. Auxiliary Substances Bearing the Phenoxy Group

**Figure 11:**
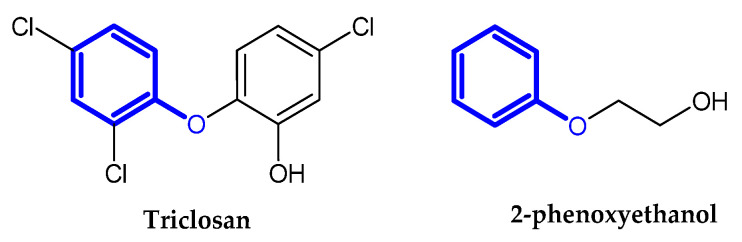
Auxiliary substances bearing phenoxy moiety.

Cosmetics, vaccines, and pharmaceuticals contain preservatives. Therefore, they keep their properties and activity longer. Triclosan and 2-phenoxyethanol are commonly used as antimicrobial ingredients [16,17]. Preservatives bearing the phenoxy moiety are presented in Figure 11.

## 3. Novel Agent with the Terminal Phenoxy Group from the Most Recent Studies

The latest developments in the field of new potential terminal phenoxy agents in 2013–2022 are presented below. The paper presents information on neurological, anti-cancer, potential lymphoma, anti-HIV, anti-microbial, anti-parasitic, analgesic, anti-diabetic as well as larvicide, cholesterol esterase inhibitors, anticoagulants or adrenergic agonists. Additionally, for selected structures, the Structure–Activity–Relationship (SAR) is also discussed.

### 3.1. Neurological Disorders

**Figure 12:**
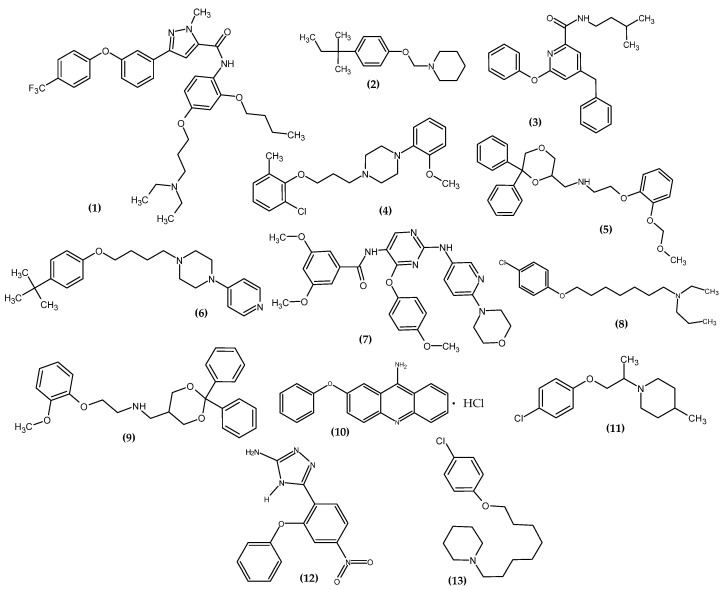
The novel potential agents for a neurological disorder bearing a phenoxy group.

In 2016, neurological disorders were the second most common cause of death [18]. On the one hand, we have depressive disorders, anxiety disorders, and phobias, and on the other, neurodegenerative diseases. The most major are Alzheimer’s and Parkinson’s [19]. An article with a meaningful title “Please remember the real me when I cannot remember you’ [20], emphasizes the need to constantly search for new potential agents for these diseases. The novel potential agents for neurological disorder bearing a phenoxy group are presented in Figure 12.

Han et al. obtained pyrazole-5-carboxamides derivatives as potent inhibitors of receptors for advanced glycation end products (RAGE) [21]. RAGE is an inflammatory factor and a critical inducer of oxidative stress, driving the development of Alzheimer’s disease [22]. The most active compound (**1**) exhibited the highest inhibitory activity against RAGE (83.1 ± 0.5%) with a half-maximal inhibitory concentration (IC_50_ = 1.9 µM). Studies of directed binding indicated that the modifications improved the binding strength with RAGE, which resulted in an increase in activity. The most active compound bore 4-trifluorophenoxy moiety [21].

Kuder et al. obtained novel tert-amyl phenoxyalkylamine derivatives targeting the histamine H3 receptor (H3R) [23]. H3R is involved in the regulation of the levels of histamine, acetylcholine, serotonin, noradrenaline, glutamate, γ-aminobutyric acid, and neuropeptide Y; therefore, it is a valuable molecular target for potential drugs for Alzheimer’s and Parkinson’s diseases, schizophrenia, narcolepsy, obesity, and the Attention Deficit Hyperactivity Disorder (ADHD) [24,25,26,27,28,29]. Compound (**2**) showed the highest affinities for human H3R in in vitro binding studies with inhibition constant (K_i_ = 8.8 nM). In the cAMP accumulation test in the HEK293 cell line, the proposed compound induced a blockade of cAMP level reduction by the receptor agonist—forskolin, which makes it an antagonist of histamine H3R with half maximal effective concentration (EC_50_ = 157 ± 16 nM). The most active compound bore 4-isopentylphenoxy moiety [23].

**Figure 13:**
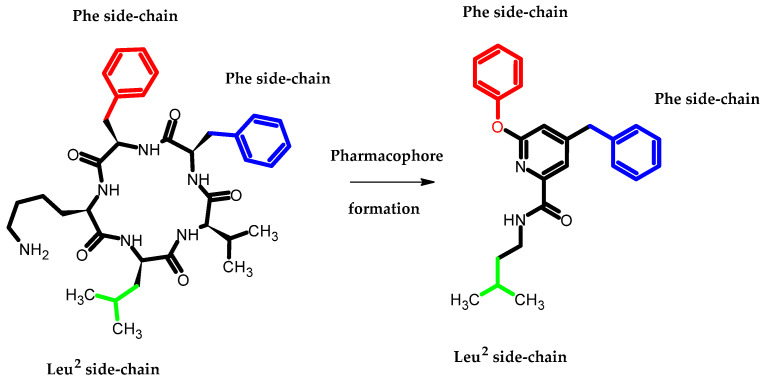
Schematic representation of the developed pharmacophore model in which the terminal phenoxy group plays the role of mimicking the phenylalanine side chain.

Arai et al., based on the Structure–Activity–Relationship (SAR) study of cyclic Aβ16-20 (cyclo-[KLVFF]), identified crucial pharmacophore elements for inhibitors of Aβ aggregation [30]. The KLVFF region is an amino acid peptide fragment of Aβ16-20, related to the formation of Aβ fibrils [31]. Therefore, its inhibition is a potential molecular target in the treatment of Alzheimer’s disease. The authors designed a non-peptide small molecule inhibitor (**3**) bearing a phenoxy group to play the role of mimicking the phenylalanine side chain (Figure 13). The compound (**3**) possessed the 71 ± 9.4% of intensity in the thioflavin-T dye assay, related to activity in inhibiting the aggregation of Aβ. The authors stated that the presence of phenoxy group is one of the three, next to the isopentyl carboxamide and benzyl groups, crucial for the activity of this compound [30].

Kubacka et al. studied the biological activity of a series of novel aryloxyalkyl derivatives of 2-methoxyphenylpiperazine in a mouse model of Major Depressive Disorder (MDD) [32]. The authors focused on the study of affinity for serotonin receptors, related to the proven role of 5-HT_1A_, 5-HT_2A_, 5-HT_3_, 5-HT_6_, and 5-HT_7_ in MDD or anxiety, as well as in antidepressant and anxiolytic effects [33,34,35,36]. They investigated the effect of substituting the phenoxy moiety with chlorine atom and methyl group in various combinations while changing the length of the alkyl chain. Compound (**4**) exhibited the most exemplary pharmacological properties in the form of partial 5-HT_1A_ receptor agonist, 5-HT_2A_ antagonist and high affinity for 5-HT_7_, K_i_ = 0.5 nM, 138.5 nM, and 34 nM, respectively. The results indicated it as an excellent potential antidepressant and anxiolytic agent. The most active compound bore 2-chloro-6-methylphenoxy moiety [32].

Del Bello et al. studied the effect of ortho substitution in the phenoxy moiety and its effect on binding to the 5-HT_1A_ receptor [37]. They designed phenoxyethanamine derivatives and investigated their properties in a mouse model of anxiety in the light/dark exploration test. The methoxymethoxy substituent (**5**) possessed the best properties. In their research, the authors exhibited that the ortho substitution of the phenoxy group with large substituents increased the proportion of hydrophobic interactions in relation to polar interactions. Moreover, the key conclusion was that the ortho substitution of the phenoxy moiety played the role of structural diversification, enabling the design of compounds that will selectively recognize 5-HT_1A_ over α_1d_-adrenoceptor and the dopamine D_2_-like receptor. The phenoxy moiety was responsible for the π–π interactions with Tyr5.39 and Phe6.52, and its ortho substituent stabilized hydrogen bonds with surrounding polar residues and enhanced hydrophobic interactions with the alkyl chains of Val3.33, Ile4.57, and Ile4.59. The engineered derivative acted as a partial agonist of 5-HT_1A_ (binding [^35^S]GTP γS 5-HT_1A_ pD2 = 9.41) and may be useful in the treatment of disorders related to this receptor. The authors noted the possibility of creating hydrogen bonds with the ether oxygen atom of the phenoxy group. Moreover, they stated that the function of the ether oxygen atom of the phenoxy moiety is presumably favoring the optimal arrangement of the phenyl ring at the binding site and is never involved in direct interactions in complexes with the open model. The most active compound bore 2-methoxy(metoxy)phenoxy substituent [37]. This underlines the privileged nature of the phenoxy moiety as being responsible for improving the interactions with the binding site.

Szczepańska et al. obtained a series of phenoxyalkylamine derivatives and examined their properties in silico, in vitro, and in vivo towards the human H3R (hH3R) [38]. The compound (**6**) turned out to be the most active, bearing a pyridin-4-yl substituent and a tert-butylphenoxy group linked by a 4-carbon alkyl chain. The novel derivative was characterized as an H3R antagonist. It did not exhibit the highest activity against hH3R in an in vitro study with K_i_ = 37.8 nM but was the only one in the mouse model to possess anticonvulsant activity in the maximal electroshock-induced seizure (MES). The authors indicated that the ether oxygen atom of the phenoxy moiety was responsible for the formation of hydrogen bonds with TYR374^6.51^. The most active compound bore the 4-tert-butylphenoxy group [38].

Farag et al. obtained a series of novel dual CSF1R/DAPK1 inhibitors [39]. The structure of new derivatives was designed based on previous research that demonstrated the privilege of the phenoxypyrimidine scaffold for CSF1R inhibitors. Inhibition of colony stimulating factor 1 receptor (CSF1R) ameliorates taupathic neuritis, and inhibition of death-associated protein kinase 1 (DAPK1) inhibits the formation of tau aggregates and exerts neuroprotective properties. A molecular simulation study indicated that the phenoxy moiety was responsible for the interaction with β3 near the hinge region. Compound (**7**) exhibited the most inhibitory activity of DAPK1 and CSF1R in an in vitro kinase assay with an IC_50_ = 1.25 µM and 0.15 µM, respectively. The most active compound bore the 4-methoxyphenoxy group [39].

Łażewska et al. obtained chlorophenoxyalkylamine derivatives as potent cholinesterases: acetyl- (AChE) and butyrylcholinesterase (BuChE) [40]. Compound (**8**) turned out to be the most active against *Electrophorus electricus* (EeAChE) with an IC_50_ = 1.93 µM and BuChE from horse serum (EqBuChE) with an IC_50_ = 1.64 µM. The effect of the chlorine substituent on the phenoxy group is not clear, but it is concluded that the para position slightly increased the activity. The Lineweaver—Burk plot indicated that the proposed compound is a nonselective AChE/BuChE inhibitor. The analysis of the binding mode by docking study exhibited that the phenyl ring of the phenoxy group is responsible for the interactions of π–π with the indole moiety of TRP279 in AChE and the residues of TYR332 in BuChE. The ether oxygen atom of the phenoxy moiety stabilized the inhibitor-AChE complex, and it was related to the formation of hydrogen bonds with the rest of TYR121, a water molecule (1159), and the protonated nitrogen atom. The most active compound bore the 4-chlorophenoxy group [40].

Franchini et al. obtained a series of diphenyl derivatives bearing the phenoxy group with potential 5-HT_1A_ receptor agonist activity [41]. Compound (**9**) exhibited the highest agonist activity for 5-HT_1A_ receptor with affinity pK_i_ 5-HT_1A_ = 8.8. In rats, the anxiolytic activity was confirmed by the Elevated Plus Maze (EPM) and Open Field tests, and the antidepressive activity was confirmed by the Forced Swim test (Porsolt). Moreover, the antinociceptive activity of the compound was confirmed in the formalin test. The most active compound bore the 2-methoxyphenoxy group [41].

Kaniakova et al. obtained 7-phenoxytacrine (**10**) [42]. The idea for modifying the tacrine was related to limit its side effects. This resulted in the production of 7-methoxy tacrine, which even reached the 3rd stage of clinical trials, but its further development was halted. This led to the search for further modifications that allowed for the introduction of the phenoxy group. In the Ellman method, the proposed compound exhibited moderate and non-selective potency towards human recombinant AChE, human plasmatic BuChE, and inhibitory activity for GluN1/GluN2B receptors through the ifenprodil binding site with an IC_50_ (AChE) = 2.4 μM, IC_50_ (BuChE) = 4.9 μM, IC_50_ (GluN1/GluN2B) = 1.7 µM, respectively. Most of the *N*-methyl-D-aspartate receptors (NMDARs) are composed of the GluN1 and GluN2A-D subunits. The authors indicated that this is a unique mechanism of action towards NMDARs relative to previous modifications, indicating a key activity of the phenoxy group. This is confirmed by the results of the docking study, which revealed that the phenoxy moiety is responsible for the preferred π–π interactions with GluN2B (Phe114) and GluN1 (Tyr109) and hydrophobic interactions with GluN1 (Thr110) and GluN2B (Ile111) residues. In the rat model of NMDA-lesion-induced damage, the development of neurodegeneration in the dorsal hippocampus was reduced [42].

Abatematteo et al. obtained novel phenoxyalkylpiperidines as a sigma-1 receptor (σ_1_R) ligand with potent anti-amnesic activity [43]. σ_1_R ligands exert antidepressant and antiamnestic activities, and exhibit neuroprotective effects in preclinical models of neurodegenerative diseases [44,45]. Based on previous research, the authors stated that the phenoxy group is one of the crucial chemical groups responsible for hydrophobic interaction for high affinity σ_1_R [46]. The most active compound (**11**) bore the 4-chlorophenoxy group and was about 10-fold more active for the σ_1_R with K_i_ = 0.34 nM, as compared to the reference standard—Pentazocine with K_i_ = 3.93 nM [43].

Navidpour et al. obtained novel 4*H*-1,2,4-triazoles as novel benzodiazepine (BZD) analogues [47]. BZD exerts its action by binding to a specific domain of the GABA_A_ receptor and acts as an anti-drug, a hypnotic, a muscle relaxant, and an anticonvulsant [48,49]. Compound (**12**) possessed about 100-fold higher affinity for a GABA_A_/benzodiazepine receptor complex with an IC_50_ = 0.03 nM, as compared to the reference standard—Diazepam with an IC_50_ = 2.4 nM. The authors indicated that phenoxy derivatives of a benzodiazepine scaffold are a new class of non-rigid structures with potent anti-seizure activity [47].

Kuder et al. obtained novel chlorophenoxyalkylamine derivatives directed towards H3R [50]. Compound (**13**) exhibited the highest affinity with K_i_ = 128 nM and was classified as a cAMP antagonist in a study with HEK293 cells with an EC_50_ = 75 nM. Moreover, the authors indicated that the position of the ether oxygen atom, i.e., the presence of phenoxy moiety, is of key importance for the high activity of this group of derivatives. It is worth noting that the substitution of the phenoxy group in the para position significantly increased the binding capacity relative to the meta position—in other words, the chlorine atom in the 4 position is the most favored in the case of the H3R binding pocket [50].

### 3.2. Anticancer Activity

**Figure 14:**
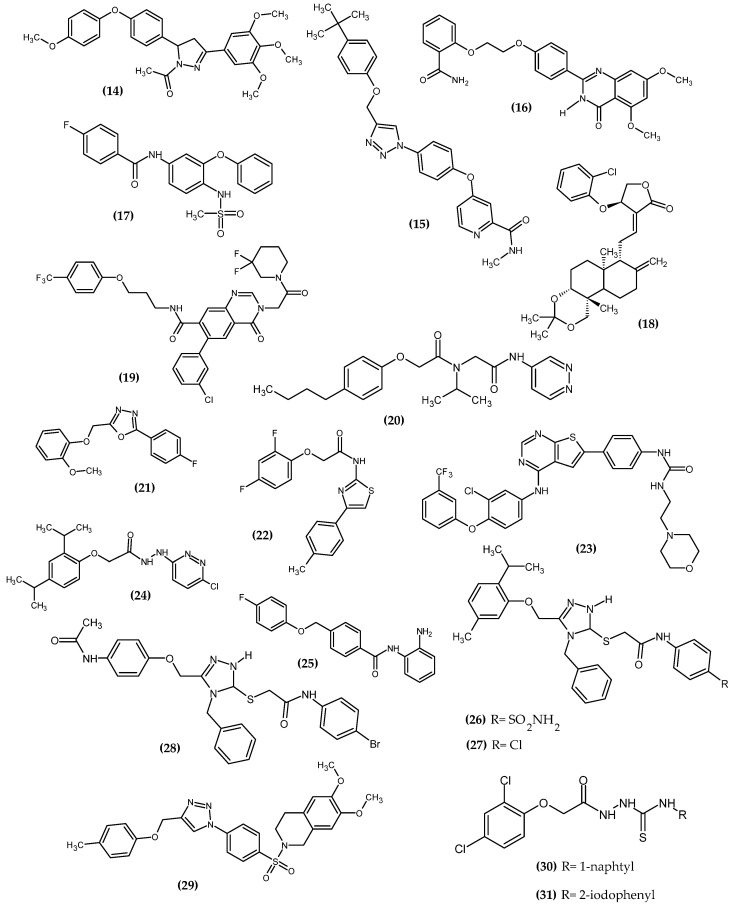
The novel potential agents with anticancer activity bearing a phenoxy group.

Cancer is one of the leading causes of death every year [51]. The American Cancer Society predicts that, in the United States alone, more than 1/3 of all newly diagnosed cancer patients will end up dying, which is about 1670 deaths a day [52]. The main factors responsible for the development of neoplasms are environmental factors resulting from human behavior. The most common ones are smoking, excessive alcohol consumption, and diet [53]. The novel potential anticancer agents bearing a phenoxy group are presented in Figure 14.

**Figure 15:**
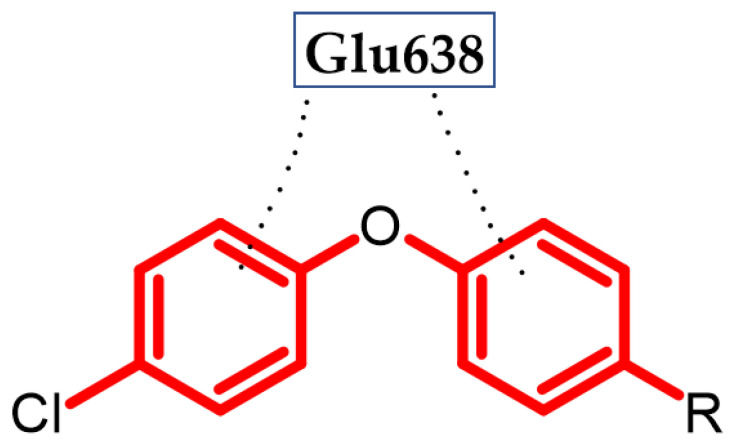
Schematic representation of H-π interactions (black dots) formed by a phenoxyphenyl moiety with STAT3 (R is the rest of the compound structure).

Hassan et al. obtained a novel series of pyrazoline derivatives as antiproliferative agents by inhibiting the vascular endothelial growth factor receptor (VEGFR) pathway [54]. Stimulation of the Janus signaling (JAK) pathway and activators of transcriptional proteins 3 (STAT3) in neoplastic cells by hypoxia and inflammatory cytokines results in the secretion of proangiogenic proteins such as vascular endothelial growth factors (VEGF). It activates the phosphorylation of its receptors (VEGFR) in endothelial cells, which leads to the induction of angiogenesis [55,56,57]. Therefore, the search for new potential agents against this signaling pathway allows for controlling tumor proliferation [58]. The basis of the design of the novel substances was the presence of the phenoxy moiety among many drugs approved by the FDA as VEGFR inhibitors, e.g., axitinib, lucytanib, and tivozanib. Compound (**14**) exhibited significant antiproliferative activity against OVCAR-4 ovarian cancer and MDA-MB-468 breast cancer cell lines with an IC_50_ = 0.29 ± 0.02 μM and 0.35 ± 0.01 μM, respectively, as compared to the reference standard—Staurosporin with an IC_50_ = 5.86 ± 0.39 µM and 3.45 ± 0.23 µM, respectively. The highest selectivity index (SI = 74) was observed for the MDA-MB-468 breast cancer cell line. The Elisa assay indicated that (**14**) reduced the VEGF concentration by 85% compared to untreated OVCAR-4 cells. Further studies indicated that the possible mechanism was inhibiting VEGFR-2 phosphorylation by 69.8%, as compared to untreated OVCAR-4 cells and by 77.5%, as compared to untreated MDA-MB-468 cells. The reason for the reduction of VEGF levels may be the inhibition of the transcription factor STAT3 [54], which was confirmed by the docking study. A docking study against STAT3 presented that phenoxyphenyl formed H–π interactions with Glu638, which are crucial for stabilizing the binding conformation (Figure 15). Cell cycle studies exhibited that compound (**14**) arrested the proliferation of the OVCAR-4 cell cycle in the S phase. It is worth noting that, for other derivatives, one of the modifications was the replacement of the phenoxy with the morpholino group, which resulted in a decrease in the activity of that compound. This underlines the impact of the phenoxy group on activity. The most active compound bore 4-methoxyphenoxy moiety [54].

Palakhachane et al. obtained novel Sorafenib analogues bearing the aryl-urea moiety of Sorafenib replaced with a 1,2,3-triazole ring linking the substituted phenoxy fragment [59]. Sorafenib is the first targeted therapy drug for patients with hepatocellular carcinoma. The authors indicated that the terminal phenoxy group could provide a hydrophobic interaction with the lipophilic pocket. Compound (**15**) was exhibited the highest activity against Huh7 hepatocellular carcinoma cell line with an IC_50_ = 5.67 ± 0.57 µM. Novel derivative (**15**) had a higher safety profile against the MRC-5 lung fibroblast cell line, as compared to the reference standard—Sorafenib. The authors noted that the substituted phenoxy group is one of the key structural elements crucial for the selective inhibition of Huh7 with a high safety profile. In other words, the phenoxy group improved the binding affinity and provided greater selectivity and less toxicity of the newer compound. The most active compound bore the tert-butylphenoxy moiety [59].

Chang et al. obtained novel quinazolin-4(3*H*)-one derivatives bearing phenoxy moiety [60]. The aim of the study was to design a bidirectional inhibitor co-targeting poly (ADP-ribose) polymerase-1 (PARP1) and protein 4-containing bromodomain (BRD4). These two targets reflect the synthetic lethal effect, having cross-links in the global breast cancer network. Compound (**16**) showed the highest activity against BD1 and BD2 with IC_50_ = 0.44 μM/L and 0.379 μM/L, respectively. Furthermore, the proposed compound (**16**) exhibited excellent inhibitory potency against BRD4 with an IC_50_ = 0.4 μM/L, as compared to the reference standard—RVX-208 with an IC_50_ = 12.6 μM/L. An antiproliferative activity study for MDA-MB-468 cells for (**16**) with an IC_50_ = 3.4–1.1 μM/L was quite lower, as compared to the reference standard—Olaparib with an IC_50_ = 1.1–1.4 μM/L. Cell cycle study indicated that (**16**) could interfere with the G1 phase, and could also induce apoptosis in a dose-dependent manner. In the SAR study, the authors stated that the phenoxy group is crucial for high activity, and it is one of the elements of the pharmacophore. The most active compound bore 2-amidophenoxy moiety [60].

Güngor et al. obtained sulfonamide containing Nimesulide derivatives [61]. From the 17th novel compound, the most active (**17**) bore the 4-fluorophenoxy group. In the sulforhodamine B assay, compound (**17**) possessed the highest activity against HT-29 colon cancer and the MCF-7 breast cancer cell line with IC_50_ = 9.24 μM and 11.35 μM, respectively. The mechanism of the antitumor effect was studied by Western blot analyses, which confirmed that expression of pro-apoptotic protein BAX (BCL-2-associated X protein) was upregulated, and anti-apoptotic protein BCL-2 (Bcell lymphoma 2) was downregulated [61].

Li et al. obtained novel andrographolide derivatives in which the 2-chlorophenoxy group was introduced at the atom C14, as a potent antibacterial agent [62]. Further investigations indicated that compound (**18**) decreased VEGF-induced phosphorylation of Akt, mTOR, MEK1/2, ERK1/2, and p38 MAPK in endothelial cells, and, by inhibiting Akt/mTOR and ERK-dependent pathways, it strongly suppressed tumor cell growth and proliferation. Furthermore (**18**), by blocking VEGFR-2-mediated signaling, it reduced VEGF expression in tumor cells and inhibited VEGF-induced endothelial cell proliferation, migration, and invasion, which is crucial for anticancer activity [63].

Ma et al. obtain a novel quinazolinone derivative as a cytosolic protein receptor 1 and 2 (NOD1/2) dual antagonist [64]. NOD1 and NOD2 are a key target for immunotherapy due to the presence of a nucleotide-binding oligomerization domain that is an important component of the innate immune system [65,66,67,68,69,70]. Antagonism of both NOD1 and NOD2 signaling guarantees the effectiveness of adjuvant cancer treatment [64]. Compound (**19**) was not the most active against both NOD1-and NOD2 in HEK293 cells with an IC_50_ = 1.13 μM and 0.77 μM, respectively; however, it possessed the highest metabolic stability. The authors stated that the oxygen atom as an ether linker connected to the phenoxy group is crucial for high activity. The most active compound bore 4-trifluoromethylphenoxy moiety [64].

Yu et al. obtained novel phenoxy derivatives as non-covalent proteasome inhibitors [71]. The ubiquitin-proteasome system is an important pathway for cell cycle progression [72,73], signal transduction [74], and immune responses [75]; thus, regulation of protease activity by inhibitors is a potential target for cancer therapy. This was confirmed by the FDA approval of bortezomib, carfilzomib, and ixazomib as proteasome inhibitors in anti-cancer therapies [71]. Compound (**20**) exhibited the highest activity against chymotrypsin-like 20S proteasome. The activity increased with the length of the alkyl chain at the para position of the PhO in the given series: methyl (481 nM) < ethyl (243 nM) < propyl (89 nM) < butyl (49 nM) (**20**). Positive control—PI-1840 possessed about 2-fold lower activity with IC_50_ = 92 ± 5 nM. Further lengthening the chain reduced the activity of the compound. The 20S proteasome consists of heptameric rings: two outer α rings and two inner β rings, and each β ring contains three proteolytic subunits β1c, β2c and β5c. Binding mode analysis indicated that the proposed compound possessed selectivity for β5c inhibitory activity with no β5i inhibition, which stated that it could be applied to solid cancers [71].

Lakshmithendral et al. obtained novel 2-(phenoxymethyl)-5-phenyl-1,3,4-oxadiazole derivatives [76]. Compound (**21**) showed the highest activity against MCF-7 and MDA-MB-453 breast cancer cell lines with an IC_50_ = 10.51 ± 1.9 μM and IC_50_ = 10.25 ± 2.5 μM, respectively. Furthermore, the proposed compound induced the apoptosis in the Acridine orange (AO)/ethidium bromide (EtBr) dual staining assay. The most active compound bore the 2-methoxyphenoxyl moiety at the 5-position and the 2-fluorophenyl moiety in the 2-position of the 1,3,4-oxadiazole ring, which determined the anti-cancer activity in the cell proliferation assays according to the authors [76].

Mohammed et al. obtained novel 4-phenyl-2-phenoxyacetamide thiazoles [77]. The trypan blue assay compound (**22**) possessed the highest activity against both MCF7 and MDA-MB 468 breast cancer cell lines, A549 lung cancer, EAC Ehrlich–Lettre ascites carcinoma, and the DLA Dalton’s lymphoma ascites cell line with IC_50_ = 14 ± 0.4 μM, 10.2 ± 1 μM, 13.2 ± 0.8 μM, 14. ± 10.2 μM, and 13.9 ± 0.4 μM, respectively. It is worth noting that the MDA-MB-468 cell lines are resistant to chemotherapy related to the lack of biomarkers; however, the proposed compound possessed quite promising activity, while the reference standard—5-Fluorouracil did not exhibit significant cytotoxicity. In the mouse EAC tumor model, the compound (**22**) reduced tumor growth and extended the life span of the animals. Furthermore, it had no apparent side effects. The most active compound bore 2,4-difluorophenoxy moiety [77].

Milik et al. obtained new thieno [2,3-d] pyrimidine-based dual EGFR/HER2 inhibitors [78]. The epidermal growth factor receptor (EGFR) family belongs to the receptor tyrosine kinases (RTKs) [79] and includes four structurally related RTKs: EGFR (HER1), HER2, HER3, and HER4 [80,81,82]. Dysregulation of EGFR related to mutations or overexpression of receptors results in excessive proliferation [83,84], resistance to apoptosis, and promotes angiogenesis and metastasis [85,86]. EGFR dysregulation is likely associated with non-small cell lung cancer (NSCLC), colorectal cancer, breast cancer, and pancreatic cancer [83,86]; thus, it is a potential target for cancer therapy. Compound (**23**) was the most active against MDA-MB-361 breast cancer and NCI-H1975 lung adenocarcinoma cancer cell lines with an IC_50_ = 3.50 ± 0.73 μM and 4.20 ± 0.19 μM, respectively, as compared to the reference standard—Lapatynib with IC_50_ = 13.73 ± 2.32 μM and 11.46 ± 2.45 μM, respectively. The most active compound bore 3-trifluorophenoxy moiety [78].

Mohammed et al. obtained novel synthesized pyridazine hydrazide appended phenoxy acetic acid [87]. Compound (**24**) exhibited the highest activity against the A549 lung cancer cell, HepG2 hepatocellular carcinoma cell, A498 kidney cancer cell line, CaSki cellosaurus cell line, and SiHa squamous cell carcinoma with IC_50_ = 6.6 ± 0.6 μM; 6.9 ± 0.7 μM; 6.8 ± 0.8 μM; 7.5 ± 0.5 μM and 7.8 ± 0.4 μM, respectively, as compared to the reference standard—5-Fluorouracil with IC_50_ = 7.4 ± 0.5 μM, 8.3 ± 1.8 μM, 5.4 ± 0.7 μM, 7.3 ± 0.4 μM, and 8.3 ± 0.7 μM, respectively. Furthermore, the proposed compound (**24**) downregulates metalloproteinase 2 (MMP-2) and metalloproteinase 9 (MMP-9) and thereby impaired metastatic cancer cell migration and invasion. The most active compound bore 2,4-diisopropylophenoxy moiety [87].

Xie et al. obtained novel 2-aminobenzamide derivatives [88]. Compound (**25**) exhibited the highest activity against the HepG2 hepatocellular carcinoma cell line with an IC_50_ = 3.84 ± 0.54 μM. The possible mechanism of anticancer activity was inducing the G2/M phase cell cycle arrest and apoptosis. Histone deacetylase (HDAC) is a promising target for cancer therapy because it is related to differentiation and apoptosis of cancer cells. Further evaluation showed two times more active potential of the proposed compound against the second isoform of HDAC with IC_50_ = 0.57 ± 0.09 μM, as compared to the reference standard—CI994 with IC_50_ = 1.20 ± 0.23 μM [87]. Compound (**25**) also possessed higher activity against the first isoform of HDAC with IC_50_ = 1.27 ± 0.20 μM, as compared to the previously mentioned standard with an IC_50_ = 1.62 ± 0.25 μM; however, its distinctly greater affinity for the second isoform is observed. The most active compound bore 4-fluorophenoxy moiety [88].

Kulabaş et al. obtained the novel 2-(4*H*-1,2,4-triazole-3-ylthio)acetamide derivatives [89]. Compound (**26**) exhibited highest activity against the PC-3 prostate cancer cell line, (**27**) against the A549/ATCC non-small cell lung cancer cell line, and (**28**) against the K-562 leukemia cancer cell line with IC_50_ = 5.96 μM, 7.90 μM, and 7.71 μM, respectively. Further studies indicated that (**26**) triggers apoptosis by using both intrinsic and extrinsic pathways, and (**27**–**28**) induce apoptotic cell death by triggering the intrinsic pathway. The most active compounds bore 5-methyl-2-(prop-2-yl)phenoxy and 4-acetylaminophenoxy moiety [89].

Pingaew et al. obtained a novel series of N-benzenesulfonyl-1,2,3,4-tetrahydroisoquinolines [90]. Compound (**29**) exhibited about 53-fold higher activity with an IC_50_ = 0.56 ± 0.01 μM against HepG2 hepatocellular carcinoma cell line, as compared to the reference standard—Etoposide with an IC_50_ = 30.16 ± 0.50. Its activity is even higher than Doxorubicin with an IC_50_ = 0.79 ± 0.08 μM. Other compounds from these series with phenoxy moiety also exhibited superior inhibitory potency toward HepG2 cells, as compared to the reference standard—Etoposide. The most active compound (**29**) bore a 6,7-dimethoxy substituent on the isoquinoline core and a p-tolyl group on the triazole moiety [90].

Gupta et al. obtained novel phenoxy thiosemicarbazide derivatives as potent antibacterial and insecticidal agent [91]. In our research, we have investigated and demonstrated anticancer activity of this compound (**30**) against the MKN74 gastric cancer cell line with an IC_50_ = 137.38 μM. Virtual screening was the basis for testing the anti-cancer potential. The proposed compound induced apoptosis by increasing the cell population in either S-phase or G2-phase. Molecular docking has shown that (**30**) acted as a DNA intercalator. The most active compound bore 1-naphtyl substituent [92]. Our last studies of 2,4-dichlorophenoxy hydrazide derivatives (**31**) revealed anti-melanoma activity on the G-361 melanoma cell line with an IC_50_ = 112 ± 4.76 μM [93]. The proposed compound was not the most active, but it exhibited a safety profile for the normal fibroblast. The possible mechanism of action downregulated the expression of dihydroorotate dehydrogenase (DHODH), which is crucial for nucleotide synthesis. Given the upregulated nature of this process as a result of proliferation, DHODH inhibitors represent a new hope for targeted therapy for melanoma [94]. Our review of the literature on the latest anti-melanoma agents revealed that the phenoxy derivatives we have obtained are among the first in recent years [95]. The most active compound bore a 2-iodophenyl substituent [93].

#### Bruton Tyrosine Kinase Inhibitors

**Figure 16:**
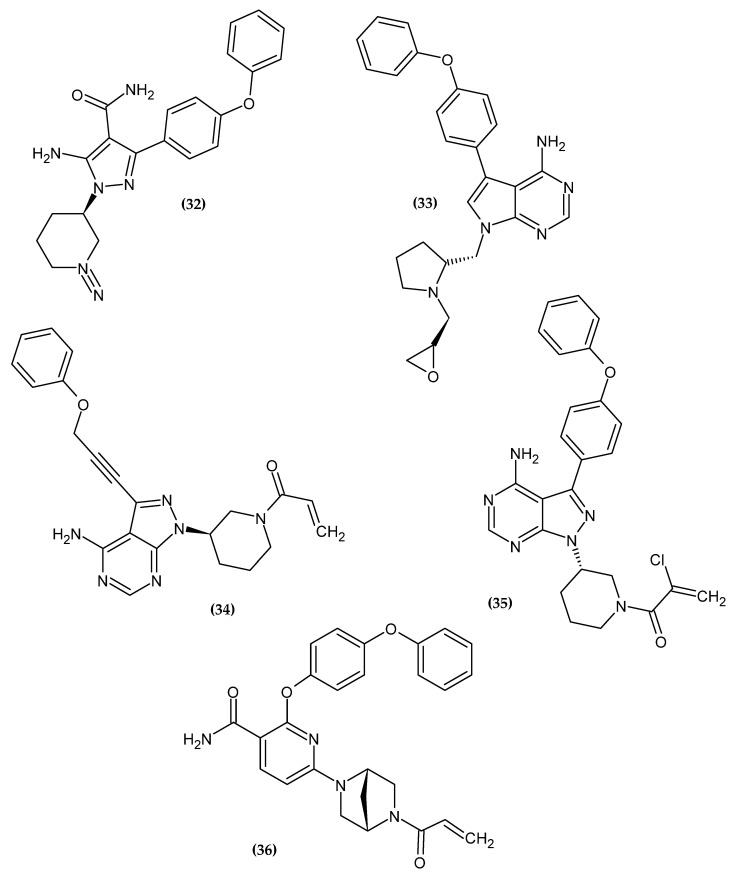
The novel potential BTK inhibitors bearing a phenoxy group.

Bruton Tyrosine Kinase (BTK) plays a key role in, among others, B-cell antigen receptor (BCR) signal transduction in normal and malignant B lymphocytes. BKT inhibitors are a new approach in the chemotherapy of chronic lymphocytic leukemia (CLL) and mantle cell lymphoma (MCL) [96]. The novel potential BTK inhibitors bearing a phenoxy group are presented in Figure 16.

Schnute et al. obtained a novel aminopyrazole carboxamide as a potent Bruton’s Tyrosine Kinase Inhibitor [97]. Compound (**32**) exhibited the best inhibitory activity against both wild type BTK and Cys481S BTK with an IC_50_ = 0.37 nM and 2.8 nM, respectively. Derivatives bearing unsubstituted phenoxy moiety had the highest activity [97].

Zhang et al. obtained novel 7*H*-pyrrolo [2,3-d]pyrimidin-4-amine derivatives as novel anti-arthritic agents [98]. Compound (**33**) exhibited the most excellent potency against Ramos and Jeko-1, the B-cell lymphoma cell lines, and Daudi BTK enhanced cell line with IC_50_ = 8.52 μM, 11.10 μM, and 7.04 μM, respectively. Furthermore, in an enzymatic assay, it possessed the highest inhibitory potential for BTK with an IC_50_ = 3.0 nM. Molecular docking revealed that phenoxy moiety is responsible for hydrophobic interaction [98].

**Figure 17:**
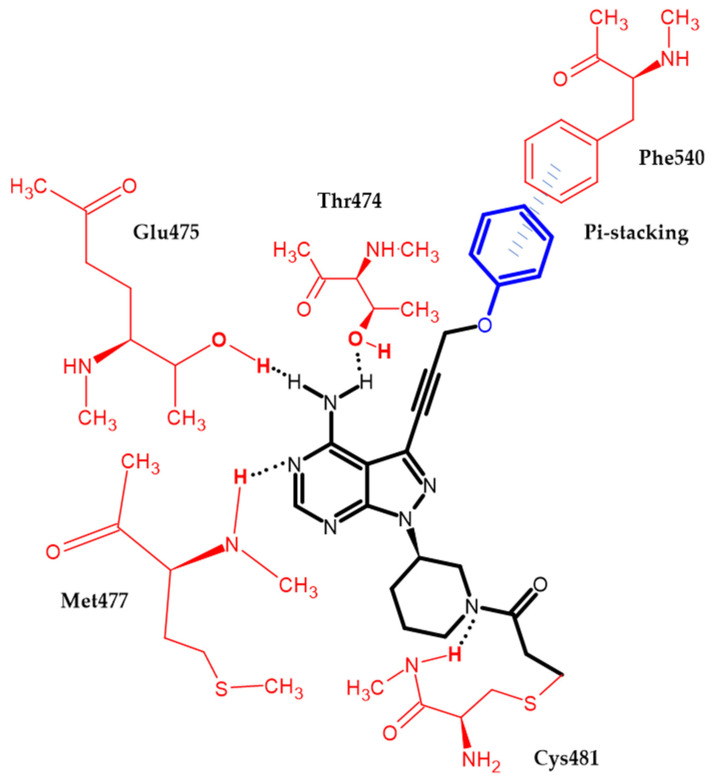
Schematic representation of the pi-stacking (blue lines) interaction formed by a phenoxy group with a BTK enzyme. The black dots represent hydrogen bonds.

Zheng et al. focused on modifying the phenyl chain linking the phenoxy group to the pyrazolopyrimidine core of Ibrutinib [99]. Modification of the elongation of this chain improves the phenoxy interaction. Compound (**34**) exhibited excellent potency Ramos and Raji for the B-cell lymphoma cell lines with an IC_50_ = 8.91 μM and 1.80 μM, respectively. Furthermore, in an enzymatic assay, it had the highest inhibitory potential for BTK with an IC_50_ = 7.95 nM. Terminal phenoxy moiety is responsible for pi-stacking interaction [99] (Figure 17).

Huang et al. showed another analog with a chlorine atom attached to the acrylamide head of Ibrutinib [100]. The proposed compound (**35**) had high activity against recombinant human BTK kinase with an IC_50_ = 2.5 nM. Furthermore, it also exhibited potent inhibitory activity against LY-10, DOHH-2, REC-1, and Mino lymphoma cell lines with IC_50_ = 0.16 μM, 0.22 μM, 0.01 μM and 0.56 μM, respectively [100].

Qiu et al. obtained a novel irreversible covalent BTK inhibitor [101]. The authors initially introduced a morpholinocarbonylphenoxy substituent into the pyridine backbone replacing the morpholinocarbonylphenylamine substituent, which resulted in a significant loss of activity, possibly due to a flip of the O-linked group into the selectivity pocket. Another modification was the introduction of a phenoxyphenoxy substituent, which resulted in a 25-fold loss of potency but significantly improved the permeability profile. The introduction of a phenoxyphenoxy group is crucial for the interaction with the selectivity pocket of the BTK kinase domain [101,102,103,104,105]. It is used as a selectivity pocket group. Knowing the beneficial effect of the phenoxyphenoxy substituent, the authors focused on modifying the linker between the core pyrimidine and ethenylcarbonyl. Compound (**36**) exhibited the best inhibitory potency in an enzymatic assay against BTK with an IC_50_ = 0.7 nM. A molecular docking study revealed that the phenoxyphenoxy group produced hydrogen bonds with Lys430 and Asp539 and occupied a selective pocket. This is another example of an active compound bearing phenoxyphenoxy moiety [101].

### 3.3. Antimicrobial Activity

The Center for Disease Control and Prevention reports that, in the United States alone, there are over 2.8 million antibiotic-resistant infections each year and 35,000 deaths [106]. There is even talk of a crisis of antibiotic therapy, related to their increasingly lower effectiveness against pathogens. The most popular are: Methicillin-resistant *Staphylococcus aureus*, Vancomycin-resistant *Enterococci*, drug-resistant *Streptococcus pneumoniae*, drug-resistant *Mycobacterium tuberculosis*, Carbapenem-resistant *Enterobacteriaceae* (CRE), multi drug resistant (MDR-spectrum) *Pseudomonas aeruginosa*, MDR *Acuginobeta*, Extended-spectrum beta-lactamase (ESBL)-producing *Enterobacteriaceae*, or drug-resistant *Neisseria gonorrhoeae* [4]. A similar problem is with antifungal drugs.

**Figure 18:**
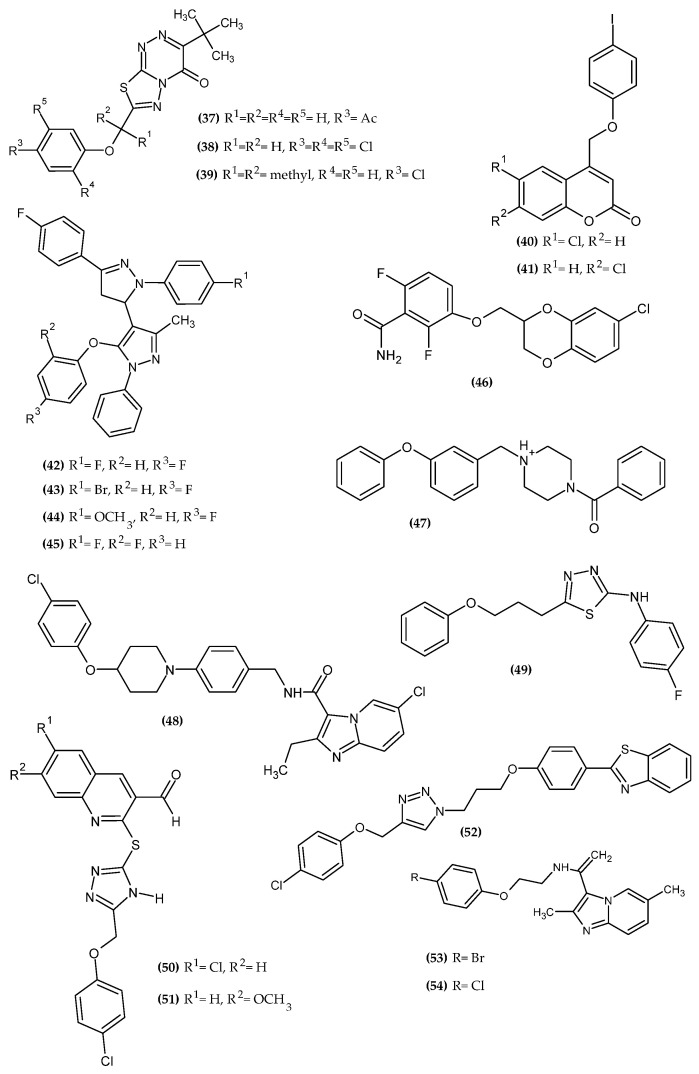
The novel potential agents with antimicrobial activity bearing a phenoxy group.

The dominance of the 4-chlorophenoxy moiety is clearly observed, which may prove its key importance for antimicrobial activity. The novel potential antimicrobial agents bearing a phenoxy group are presented in Figure 18.

Castelino et al. obtained a series of novel thiadiazolotriazin-4-ones as a potent antibacterial agent [107]. Compound (**37**) exhibited the highest activity against *B. subtilis* and *P. aeruginosa* with 25.7 ± 0.65 mm and 22.7 ± 0.68mm, (**38**) against *S. aureus* with 22.4 ± 0.69 mm and (**39**) against *K. pneumoniae* with 22.4 ± 0.65mm zone of inhibition (compounds concentration 1 μg/mL), respectively, as compared to the reference standard—Streptomycin (10 μg/disc) with 33.6 ± 0.78 mm, 26.3 ± 1.08 mm, 31.5 ± 0.84 mm, and a 22.4 ± 0.79 mm zone of inhibition for given bacteria species, respectively. The authors found that the presence of a phenoxy group in the para position of the thiadiazolotriazine scaffold provided a broad spectrum of antibacterial activity for both Gram-positive and Gram-negative species. The most active compounds bore 4-acetylphenoxy, 2,4,5-trichlorophenoxy and 4-chlorophenoxy moiety [107].

Basanagouda et al. obtained a series of novel iodinated-4-aryloxymethylcoumarins with promising activity against *Mycobacterium tuberculosis* [108]. The most active compounds were (**40**–**41**), and they both exhibited the same activity against *Mycobacterium tuberculosis* H37 RV with minimum inhibitory concentration—MIC = 1.56 μg/mL and *Mycobacterium phlei* with MIC = 3.125 μg/mL, as compared to the reference standards—Streptomycin and Pyrizanamide with MIC = 6.25 μg/mL and 3.125 μg/mL, respectively. Both compounds were also screened for anticancer activity and also possessed significant cytotoxicity effects. The authors found that, for an iodine substituent, anticancer and antituberculosis activity increased in a given series: orto > meta > para in the phenoxy group [108].

Karad et al. obtained a novel series of fluoro substituted pyrazolylpyrazolines as antimicrobial agents [109]. Compound (**42**) exhibited the highest activity against *S. pneumoniae* and *C. albicans* with MIC = 100 μg/mL and 200 μg/mL, respectively, as compared to the reference standard—Ampicillin and Griseofulvin with MIC = 100 μg/mL and 500 μg/mL, respectively. Compound (**43**) was the most active against *C. tetani* with MIC = 125 μg/mL and compound (**44**) against *B. subtilis* with MIC= 62,5 μg/mL, as compared to the reference standard—Ampicillin with MIC = 250 μg/mL for both given bacterial strain. Compound (**45**) showed the highest activity against *A. fumigatus* with MIC = 100 μg/mL, as compared to the reference standard—Nystatin with MIC = 100 μg/mL. Furthermore, Compound (**43**) had 96% of inhibition (at concentration 250 μg/mL) against *M. tuberculosis* H37 RV, as compared to the reference standards—Rifampicin and Isoniazid with 98% and 99% of inhibition, respectively. Screening against antimalarial activity indicated that (**42**) with an IC_50_ = 0.022 μg/mL is comparable to the reference Chloroquine with an IC_50_ = 0.020 μg/mL and has superior potency to Quinine with an IC_50_ = 0.268 μg/mL. The authors stated that the 4th position of the substituent of the phenoxy group, especially for fluorine, is crucial for antimalarial and antituberculosis activity [109].

Chiodini et al. obtained a novel series of 3-substituted 2,6-difluorobenzamides for potent bacterial cell division inhibition [110]. Compound (**46**) was the S-enantiomer and possessed antibacterial activity against *S. ureus* with MIC = 0.25 μg/mL and minimum bactericidal concentration—MBC = 0.5 μg/mL, as compared to the reference standard—2,6-difluoro-3-nonyloxybenzamide with MIC = 0.1 μg/mL and MBC = 0.25 μg/mL. The most active compound bore 3-amido-2,4-difluorophenoxy moiety [110].

Kanetaka et al. obtained a novel potential *M. tuberculosis* enoyl acyl-carrier protein reductase (mtInhA) inhibitor through matched molecular pair analysis and in silico screening [111]. MtInhA is a validated target enzyme for tuberculosis treatment [112]. It is worth mentioning that all of compounds chosen by an in silico methods, bearing a phenoxy group, and taking into account the role of a diphenyl ether ring in the binding site, this element was a privileged part of a scaffold for this activity. Compound (**47**) exhibited the highest activity against mtInhA with an IC_50_ = 12 µM, as compared to the reference standard with an IC_50_ = 9.8 µM [111].

Kang et al. obtained Telacebec (Q203) analogues with different side chains and studied their effects on anti-tubercular activity [113]. Compound (**48**) exhibited the highest activity with extracellular minimum concentration required to inhibit growth by 80%—MIC_80_ = 3 nM, as compared to the Q203 with MIC_80_ = 4 nM. The derivative bearing a chlorine atom in the para position of the phenoxy moiety was the most active [113].

Muğlu et al. obtained novel 1,3,4-thiadiazole compounds derived from 4-phenoxybutyric acid [114]. Compound (**49**) exhibited the best antibacterial activity (final concentration = 5 μg/μL) against *S. aureus* with given inhibition zone: 17 mm at 30 µL, 18 mm at 50 µL, and 20 mm at 80 µL. The most active compound bore an unsubstituted phenoxy group [114].

D’Souza et al. obtained novel cyclic 4-chlorophenoxy thiosemicarbazide derivatives for potent antibacterial and antifungal activity [115]. Compound (**50**) possessed about 2-fold higher activity against *E. coli*, *S. aureus*, *P. aeroginosa*, and *K. pneumonia* with MIC = 3.125 µg/mL for each given strain, as compared to the reference standard—Ampicillin with 6.25 µg/mL for all mentioned bacterial stains. Furthermore, Compound (**50**) exhibited activity against *P. marneffei*, *T. mentagrophytes*, *A. flavus*, and *A. fumigatus* with MIC = 6.25 µg/mL for all given species of fungi, as compared to the reference standard—Itraconozole with MIC = 6.25 µg/mL. Compound (**51**) also exhibited great activity against *P. marneffei* and *A. flavus* with the same MIC values as (**50**). Both of the most active derivatives bore 4-chlorophenoxy moiety [115].

**Figure 19:**
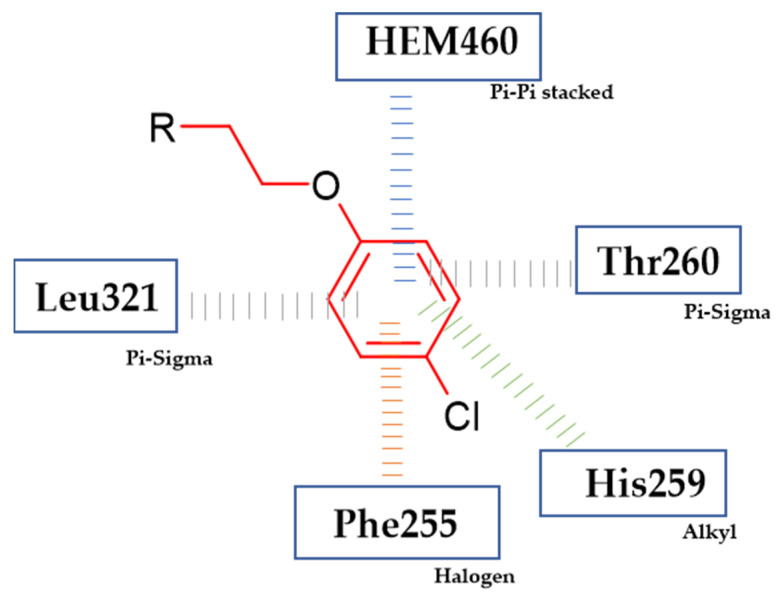
Schematic representation of the hydrophobic interactions (lines) formed by a phenoxy group with an active site of cytochrome 450 14α-sterol demethylase enzyme CYP51 (R is the rest of the compound structure).

Nehra et al. obtained novel bioactive 1,2,3-triazole hybrids [116]. Compound (**52**) exhibited antifungal activity (final concentration = 50 µg/mL) against *C. tropicalis* and *A. terreus* with 33.1 mm and 30.5 mm zone of inhibition, respectively, as compared to reference standard—Fluconazole with 21 mm and 19 mm zone of inhibition, respectively. A molecular docking study revealed that phenoxy moiety is responsible for hydrophobic interaction (Figure 19). The most active compound bore 4-chlorophenoxy moiety [116].

Wu et al. obtained novel N-(-2-phenoxyethyl)imidazo [1,2-a]pyridine-3-carboxyamides [117]. Compound (**53**) exhibited the highest activity against *M. tuberculosis* H37 RV with MIC = 0.027 μg/mL, as compared to the reference standards—Isoniazid and Rifampicin with MIC = 0.049 μg/mL and 0.05 μg/mL, respectively. Furthermore, compound (**54**) revealed great activity against MDR-MTB 11168 and MDR-MTB 9160 with MIC = 0.025 μg/mL and MIC = 0.028 μg/mL, respectively. It is worth noting that both strains are resistant to Isoniazid and Rifampicin. The authors stated that the oxyethyl linker from the phenoxy group possessed better activity than the aminoethyl one. The most active compounds bore 4-brominephenoxy and 4-chlorophenoxy moiety [117].

### 3.4. Anti-HIV Activity

According to UNAIDS data, more than 36 million people have died from AIDS-related diseases since the epidemic began. In 2020 alone, over 1.5 million people became infected with HIV [118]. This indicated an urgent need to search for new potential drugs for this virus.

**Figure 20:**
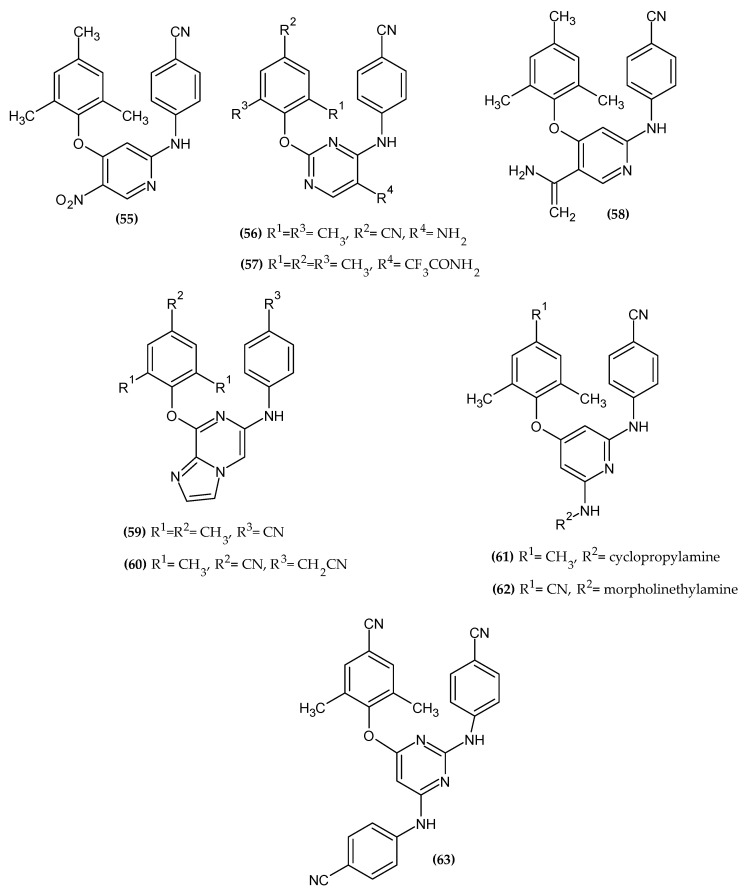
The novel potential agents with anti-HIV activity bearing a phenoxy group.

The dominance of the 4-substituted-2,6-dimethylphenoxy moiety is clearly observed, which may prove its key importance for anti-HIV activity. The novel potential anti-HIV agents bearing a phenoxy group are presented in Figure 20.

Wang et al. obtained novel nitropyridine derivatives [119]. Compound (**55**) did not exert the highest activity against MT-4 cells infected by wild-type HIV-1 strain III_B_ with an EC_50_ = 0.056 μM but possessed the best selectivity index (SI = 1251). It is about 2-fold higher than the highest activity compound from reported series. Even if it was not the most active, it still has significant activity compared to Nevirapine and Delaviridine with an EC_50_ = 0.23 μM and 0.51 μM, respectively. The activity of proposed compound (**50**) was also tested against wild-type HIV-1 reverse transcriptase (RT) with an IC_50_ = 6.9 μM, which is comparable to the reference standard—TMC125 with an IC_50_ = 6.5 μM. Reverse transcriptase enzyme is responsible for the catalytic synthesis of viral DNA from the viral ssRNA genome, which enables replication; therefore, it is a valuable molecular target for therapy [120]. The authors also investigated the replacement of the ether oxygen of the phenoxy moiety with the amino group, obtaining derivatives with a significantly lower anti-HIV potency, which emphasizes the key presence of the phenoxy group for the desired biological activity. The authors indicated that the phenoxy group from the pharmacophore model is responsible for the π–π interactions. The most active derivative bore 2,4,6-trimethylphenoxy moiety [119].

Li et al. obtained novel diarylpyrimidines [121]. Authors found that the presence of the phenoxy group is crucial for forming an important hydrophobic interaction with an HIV-1 non-nucleoside RT binding pocket. Compound (**56**) possessed moderate anti-HIV-2 activity with an EC_50_ = 5.57 μM, and compound (**57**) exhibited the highest activity against HIV-1 wild-type and double RT mutant HIV-1 strain K103N/Y181C, with an EC_50_ = 0.002 ± 0.001 μM and 0.33 µM, respectively, as compared to the reference standard—Nevirapine with an EC_50_ = 0.22 ± 0.06 μM and 4.0 ± 3.7 μM, respectively. Proposed compound (**57**) turned out to be the most selective compared to previously mentioned targets. The most active derivatives bore the 4-cyano-2,6-dimethylphenoxy and 2,4,6-trichlorophenoxy moiety [121].

**Figure 21:**
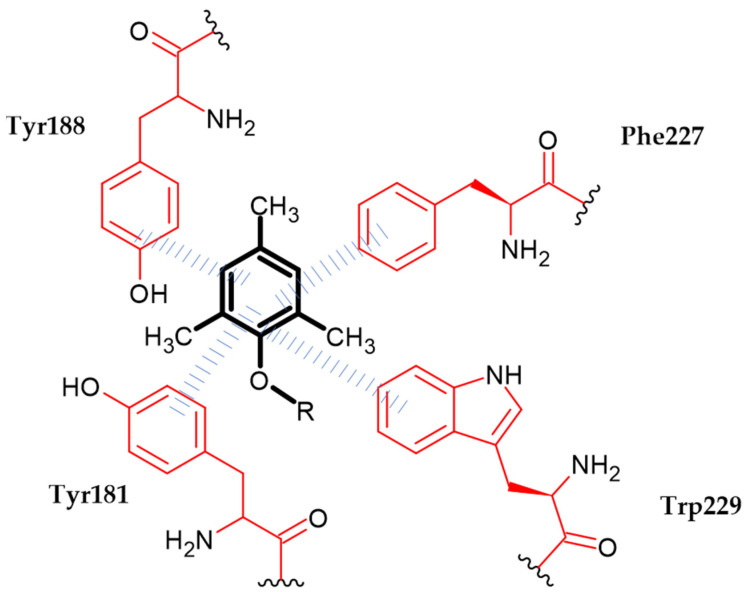
Schematic representation of the π–π interaction (blue lines) formed by a phenoxy group (R is the rest of the compound structure).

Liu et al. obtained a series of novel diarylnicotinamide derivatives targeting the entrance channel of HIV-1 non-nucleoside RT [12]. Authors stated that the phenoxy group is one of three crucial elements of scaffolds for antiviral compounds. Compound (**58**) exhibited highly potent inhibitory activity against HIV-1 replication with an EC_50_ = 0.027 µM and significant SI > 12,518, as compared to Nevirapine with an EC_50_ = 0.31 ± 0.015 µM. In enzymatic assays against HIV-1 RT wild type, the proposed compound possessed comparable activity with an EC_50_ = 0.02 ± 0 µM to the reference standard—Nevirapine with an EC_50_ = 0.02 ± 0.002 µM. Molecular docking study indicated that 2,4,6-trimethylphenox-1-yl group is responsible for π–π interaction with aromatic amino acid residues Tyr181, Tyr188, Phe227 and Trp229 (Figure 21). The most active compound bore 2,4,6-trimethylphenoxy moiety [12].

Huang et al. obtained a series of novel imidazo [1,2-a]pyrazine derivatives as HIV-1 non-nucleoside RT inhibitors [122]. Compound (**59**) possessed the highest activity against MT-4 cell cultures infected with the wild-type HIV-1 III_B_ with an EC_50_ = 0.26 µM, as compared to Nevirapine with an EC_50_ = 0.31 µM in a cell-based assay. The enzymatic assays against HIV-1 RT wild type demonstrated the highest potency of compound (**60**) with an IC_50_ = 0.17 µM, as compared to the reference standard—Etravirine with an IC_50_ = 0.13 µM. The most active compounds bore 2,4,6-trimethylophenoxy and 4-cyano-2,6-dimethylphenoxy moiety [122].

Yang et al. obtained novel diarylpyridine derivatives targeting the entrance channel of HIV-1 non-nucleoside RT [11]. Compound (**61**) possessed the highest activity against wild-type HIV-1 with an EC_50_ = 35 nM, as compared to the reference standard—Delavirdine with an EC_50_ = 33 nM. Furthermore, antiviral activity of compound (**62**) against the K103N mutation was about 140-fold higher with an EC_50_ = 49 nM, as compared to the reference standard—Nevirapine with an EC_50_ = 6.78 µM. Authors stated that substituted phenoxy moiety is a crucial part of pharmacophore responsible for hydrophobic interaction. The most active compounds bore 2,4,6-trimethylphenoxy and 4-cyano-2,6-dimethylphenoxy moiety [11].

Meng et al. obtained novel diarylpirimidine derivatives as a potent HIV-1 non-nucleoside RT inhibitor [123]. Compound (**63**) exhibited the highest activity with an EC_50_ = 0.11 µM and 2.18 µM against HIV-1 III_B_ and K103N/Y181C double mutant HIV-1 strain (RES056), respectively, as compared to the reference standard—Nevirapine with an EC_50_ = 0.28 ± 0.038 μM and >15.02 μM, respectively. Inhibitory activity of (**63**) against HIV-1 RT was over 100-fold more potent with an IC_50_ = 0.0727 μM, as compared to the reference standard—Nevirapine with an IC_50_ = 8.8 μM. The cyano group at the para position of the phenoxy group is crucial for high activity. Authors stated that phenoxy moiety is responsible for hydrophobic interaction. The most active compound bore 4-cyano-2,6-dimethylphenoxy moiety [123].

### 3.5. Antiparasitic Activity

**Figure 22:**
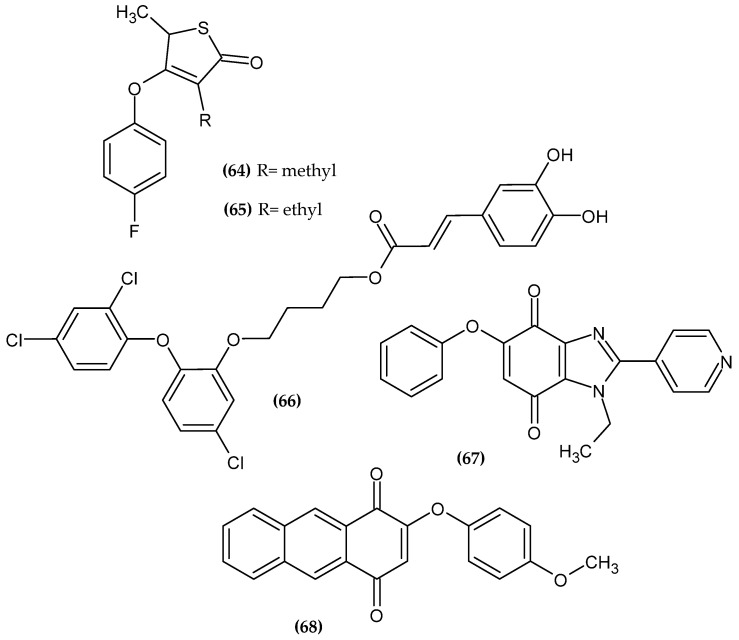
The novel potential agents with anti-parasitic activity bearing a phenoxy group.

The Anopheles mosquito transmits Plasmodium parasites that cause malaria. Estimates from 2017 showed that over 219 million people were affected by malaria, and over 400,000 died [124]. Another parasitic disease is Human Trypanosomiasis, otherwise known as coma, caused by *Trypanosoma brucei*. If left untreated, the disease is almost always fatal [125]. The novel potential antiparasitic agents bearing a phenoxy group are presented in Figure 22.

Sainy and Sharma obtained novel thiolactone derivatives as a novel antimalarial agent [126]. Compound (**64**) turned out to be the most active against Chloroquine resistant strain *P. falciparum* with an IC_50_ = 0.09 µM and (**65**) against Chloroquine—sensitive strain *P. falciparum* with an IC_50_ = 0.03 µM, as compared to the reference standard—Chloroquine with an IC_50_ = 0.20 µM and 0.03 µM, respectively. Compound (**65**) also had the same high activity against the last target as (**64**). These results were confirmed by molecular docking to β-keto acyl −ACP synthase I/II (KAS I/II), which is responsible for the synthesis of fatty acids. They are crucial for the membrane biosynthesis, which is necessary for the formation of the invasive stage. Therefore, this enzyme represents a promising target for new antimalarial drugs. Furthermore, authors stated that the para-substituted phenoxy group is stabilized by hydrophobic interactions with active site residues of the enzyme. The fluorine atom in the para position of the phenoxy moiety is found in the most active compounds [126].

Otero et al. obtained novel Triclosan-caffeic acid hybrids [127]. Proposed compound (**66**) exhibited anti-leishmanial activity against *L. (V.) panamensis* and *T. cruzi* with an EC_50_ = 3.82 ± 0.19 μM and 8.25 ± 1.21 μM on intracellular amastigotes, as compared to the reference standard—Triclosan and Benznidazole with an EC_50_ = 38.61 ± 2.38 μM and 40.3 ± 6.92 μM, respectively. The most active compound bore 2-(2,4-dichlorophenoxy)-5-chlorophenoxy moiety [127].

López-Lira et al. obtained novel benzimidazolequinones as potent trypanosomicidal agents [128]. Proposed compound (**67**) had high activity against epimastigote and trypomastigote forms of *T. cruzi* (Dm28c strain) with an IC_50_ = 1.42 ± 0.07 μM and 0.65 ± 0.10 μM, respectively, as compared to the reference standard—Nifurtimox with an IC_50_ = 21.05 ± 0.90 μM and 10.00 ± 0.40 μM, respectively. Authors stated that phenoxyquinones can be considered as new anti trypanosomical agents with lower cytotoxicity against normal cells. The most active compound bore phenoxy moiety [128].

Prati et al. obtained novel 2-phenoxy-1,4-anthraquinone derivatives [129]. Compound (**68**) exhibited the highest activity against the clinically relevant form of *T. brucei* with an IC_50_ = 0.38 μM. The introduction of electron donating groups such as NHAc, CH_3_, and the OCH_3_ to the phenoxy group resulted in increased stability of the derivatives. The methoxy substituent in the para position of the phenoxy moiety is found in the most active compound [129].

### 3.6. Analgesic Activity

**Figure 23:**
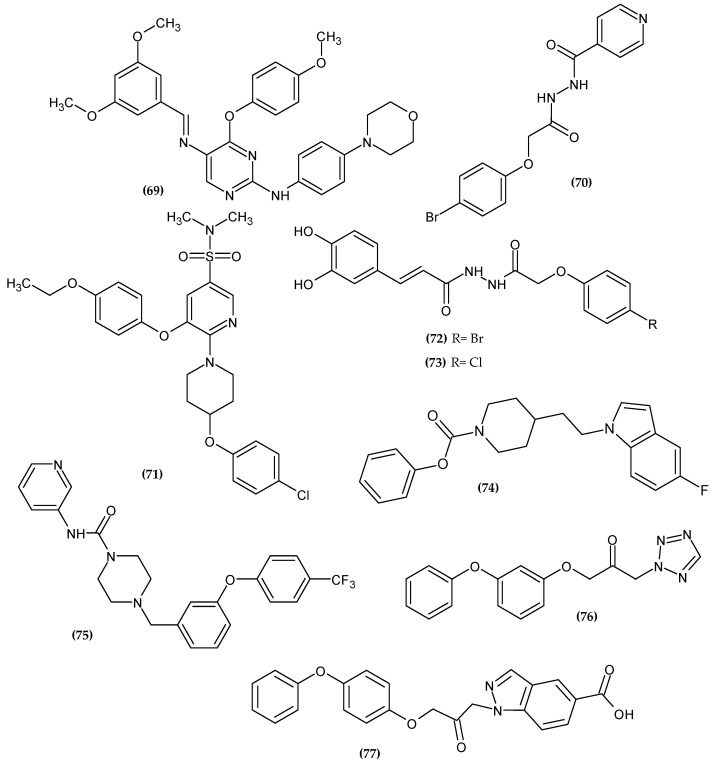
The novel potential agents with analgesic activity bearing a phenoxy group.

Pain is one of the most common symptoms that patients seek medical attention for. Despite the breakthrough discovery of opioids, salicylic acid, etc. several decades ago, first-line drugs still do not have long-term efficacy, e.g., long-term opioid use results in hyperalgesia. The complexity of the pain pathomechanism is a major challenge for medical chemists, so it is imperative to continue looking for potential drug agents that will provide a response to this problem [130]. The novel potential analgesic agents bearing a phenoxy group are presented in Figure 23.

Farag et al. obtained a novel colony-stimulating factor receptor (CSF1R) inhibitor based on a phenoxypyrimidine scaffold [131]. CSF1R is mainly expressed in macrophages, making it a suitable target for macrophage-induced inflammatory diseases. Compound (**69**) was selectively potent over a mentioned target with an IC_50_ = 4.6 nM. Authors stated that an orto- or meta- phenoxy group substituent capable of forming hydrogen bonds is essential for CSF1R activity. The methoxy group at the 4-position of the phenoxy group (**69**) formed a hydrogen bond with Lys612. The compound that bore 4-methoxyphenoxy moiety was the most active [131].

**Figure 24:**
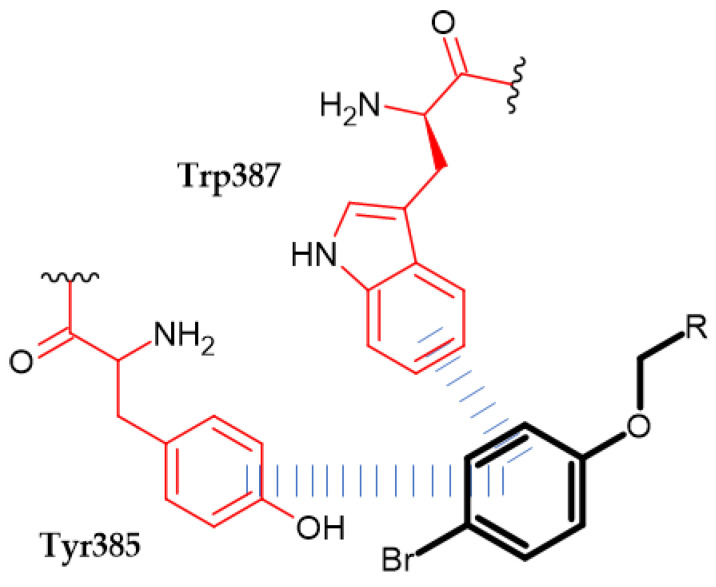
Schematic representation of the π–π interaction (blue lines) formed by a phenoxy group (R is the rest of the compound structure).

Pallavi et al. obtained novel N-(2-phenoxyacetyl) isonicotinohydrazide derivatives as anti-inflammatory and analgesic agents [132]. Cyclooxygenases 1 and 2 (COX-1 and COX-2) are key enzymes for the maintenance of health and injury. COX is involved in the biosynthesis of prostaglandins (PG) and thromboxane (TX), which mediate pain and inflammation. Therefore, inhibiting COX exerts an analgesic and anti-inflammatory effect [133]. Compound (**70**) possessed the most promising activity against COX-1 and COX-2 with an IC_50_ = 11.107 µM and 0.095 µM, respectively. Furthermore, it exhibited the highest COX-2 selectivity index (SI = 116.91). Authors stated that bromo substituent at the para position at phenoxy moiety improved selectivity index, and it was found in the most active compound. A molecular docking study of binding (**70**) with COX-2 revealed π–π interaction between the phenoxy group and TYR385 and TRP387 [132] (Figure 24).

Carrasco et al. obtained novel 2,3,5-trisubstituted pyridine analogs as potent inhibitors of Interleukin-1β (IL-1β) [134]. IL-1β is an inflammatory mediator that is released in response to injury [135,136,137]. Its excessive production is associated with the pathogenesis of autoinflammatory diseases [138]. Monotherapy with an IL-1β inhibitor quickly and permanently reduces the severity of the disease, thus it is a valuable molecular target. Compound (**71**), which bore a 4-ethoxyphenoxy substituent, was found to be the most active with an IC_50_ = 28 nM. Furthermore, compound (**71**) was also tested in vivo in a Lipopolysaccharide endotoxic shock model in male BALB/c mice, which resulted in a decreasing level of IL-1β in plasma, brain, liver, and lung tissue [134].

Al-Ostoot et al. obtained novel caffeic acid analogous as potent anti-inflammatory agents [139]. Derivatives were evaluated for an in-vivo anti-inflammatory activity by adopting the carrageenan-induced rat paw edema method [140]. Compound (**72**) exhibited 75.17 ± 4.37% and 72.29 ± 3.80% of inhibition anti-inflammatory activity after 3 and 4 h, respectively, and 67.85 ± 2.30% of inhibition of analgesic activity in the mouse model. The SAR study indicated that halogen substituents such as bromo or chloro atom at the para position of phenoxy moiety proved to be crucial for high biological activity and the selectivity index. Compound (**73**) exhibited the highest activity against COX-I with an IC_50_ = 232.68 µM and SI = 68.43. A molecular docking study revealed that phenoxy moiety is responsible for π–π stacking with TYR355 in COX-1 [139].

Fatty acid amide hydrolase (FAAH) is an important enzyme in endocannabinoid metabolism that inactivates anandamide by splitting it into arachidonic acid and ethanolamine. Thus, it interrupts the anesthetic and anti-inflammatory effect, making FAAH inhibitors a new class of painkillers and anti-inflammatory agents [141].

Dahlhaus et al. obtained (indolylalkyl)piperidine carbamates as FAAH inhibitors [141]. Compound (**74**) possessed the highest inhibitory potency against FAAH with an IC_50_ = 0.046 μM, as compared to the reference standard—URB597 with an IC_50_ = 0.06 μM. The most active compound bore unsubstituted phenoxy moiety [141].

Keith et al. obtained a series of heteroaryl piperazinyl-urea as FAAH inhibitors [142]. Compound (**75**) exhibited the highest activity against human FAAH with an IC_50_ = 1.0 ± 0.5 nM. The most active compound bore 4-trifluorophenoxy moiety [142].

Sundermann et al. obtained novel 1-heteroaryl-3-phenoxypropan-2-ones acting as inhibitors of FAAH [143]. Compound (**76**) exhibited the highest activity against the FAAH receptor with an IC_50_ = 0.008 μM. Related to the metabolic study, the fenoxycarbamate moiety was supposed to prevent the compound from being quickly deactivated in the body. The most active compound bore 2-(phenoxy)phenoxy moiety [143].

Althaus et al. obtained novel 1-(5-carboxyindazol-1-yl)propan-2-ones as FAAH inhibitors [144]. Compound (**77**) exhibited the best inhibitory activity against FAAH with an IC_50_ = 0.055 μM. The most active compound was bore a 4-(phenoxy)phenoxy group [144]. Compounds (**76**) and (**77**) possessed significantly higher activity than other FAAH inhibitors previously presented, which may be related to phenoxyphenoxy moiety.

### 3.7. Anti-Diabetic Activity

**Figure 25:**
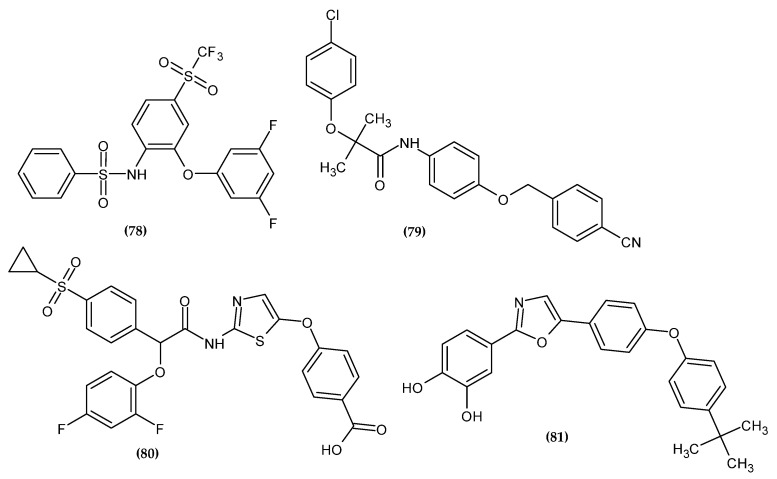
The novel potential agents with anti-diabetic activity bearing a phenoxy group.

Diabetes is characterized by a disturbance in the secretion or action of insulin resulting in hyperglycemia. Uncontrolled diabetes can lead to a coma and even death from ketoacidosis [145]. WHO reports that 422 million people worldwide suffer from diabetes [146]. The novel potential anti-diabetic agents bearing a phenoxy group are presented in Figure 25.

Desai et al. obtained a novel 1,3-difluorophenoxy derivative with an inhibitory activity of neutral amino acid transporter B0AT1 [147]. B0AT1 is the major transporter of neutral apical amino acids [148]. Mice with B0AT1 knockout have been shown to possess better glycemic control and resistance to a high-fat diet; thus, it is a potential target for the treatment of diabetes and improving metabolic health in obese subjects [149,150]. Compound (**78**) exhibited the highest activity with an IC_50_ = 0.035 μM. In the diet induced obese (DIO) mice model, the proposed compound resulted in a significant improvement in insulin tolerance and a decrease in body weight. The most active compound bore 3,5-difluorophenoxy moiety [147].

Sun et al. synthesized novel adenosine monophosphate-activated protein kinase (AMPK) activators as a potential therapeutic approach to metabolic diseases [151]. AMPK participates in the energy balance modulation process. AdipoRon activates AMPK via the adiponectin 1 receptor. However, it also inhibits mitochondrial complex I, which may result in the development of lactic acidosis. Therefore, there is a need to design new derivatives devoid of this effect. Compound (**79**) exhibited superior activity in stimulating glucose consumption (GC = 151.8 ± 4.0%). Compounds with the phenoxy moiety were generally more active than the series with the 4-aminopiperidinyl moiety. Moreover, the proposed compound had no inhibitory activity against mitochondrial complex I and did not cause the cardiac hypertrophy characteristic of this group of compounds. This proves it has a higher safety profile. The most active compound bore 4-chlorophenoxy moiety [151].

Deshpande et al. obtained novel 2-phenoxy-acetamide derivatives as glucokinase activators [152]. The glucose-phosphorylating enzyme glucokinase (GK) plays a key role in glucose homeostasis. In humans, natural KG mutations are responsible for hyper or hypoglycemia. GK deficiency in mice has been shown to result in hyperglycemia [153] and overexpression leading to improved glucose tolerance [154,155,156]. Therefore, the use of GK activators could reduce the hyperglycemia of type 2 diabetes mellitus. Compound (**80**) possessed the highest potency for recombinant human glucokinase activation with an EC_50_ = 0.034 ± 0.01 μM. Studies in mice indicated a beneficial effect on glucose metabolism with a reduced risk of hypoglycemia. The most active compound bore 2,4-difluorophenoxy moiety [152].

Li et al. obtained novel catechol derivatives as selective inhibitors of protein tyrosine phosphatases 1B (PTP1B) [157]. PTP1B are a negative regulator of the insulin and leptin signaling pathways [158,159], which makes them involved in the modulation of glucose and lipid metabolism [160,161]. Therefore, it is a promising molecular target in the treatment of type 2 diabetes and obesity. The most potent inhibitor (**81**) exhibited an IC_50_ = 0.487 μM against PTP1B. The authors noted that the hydrophobic tail of the phenoxy moiety allowed for high selectivity with good membrane permeability and provided novel class of non-insulin-dependent drugs in the treatment of type 2 diabetes. The most active compound bore 4-tert-butylphenoxy moiety [157].

### 3.8. Other Activity

**Figure 26:**
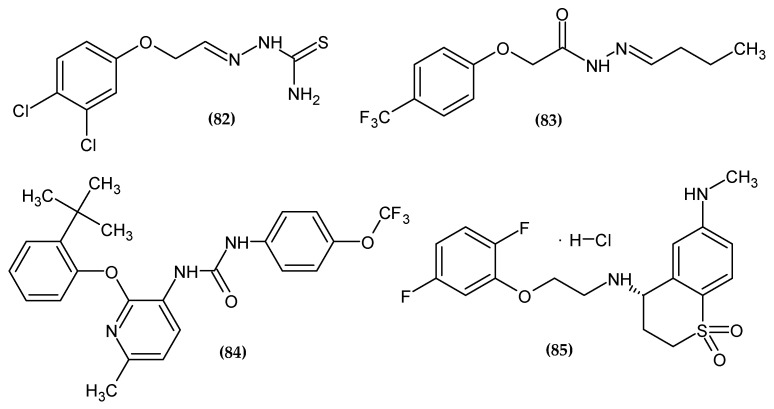
The novel potential agents with other activity bearing a phenoxy group.

Phenoxy derivatives also had larvicidal and anti-creep activity. Within this group of derivatives, cholesterol esterase inhibitors and adrenergic receptor agonists are known. The agents with previously mentioned biological activity, bearing a phenoxy group, are presented in Figure 26.

Da Silva et al. obtained a series of phenoxymethyl-thiosemicarbazones as potent larvicidal agents [162]. Compound (**82**) possessed the highest larvicidal activity with a half-maximal lethal dose (LC_50_ = 5.8 μM/L). A double-chlorine compound, especially as a meta and para substituent of phenoxy moiety, significantly increased the activity [162].

Zhao et al. obtained a selective cholesterol esterase (CEase) inhibitor [163]. Proposed compound (**83**) exhibited inhibitory effects against CEase with an IC_50_ = 0.36 μM. A molecular docking study revealed π–π interaction between phenoxy moiety and residues Trp227 and Phe324. The most active compound bore a 4-trifluoromethylphenoxy group [163].

Peng et al. obtained novel 2-(phenoxyaryl)-3-urea derivatives as novel P2Y_1_ receptor antagonists [164]. Adenosine 5′-diphosphate (ADP) plays a key role in the activation of platelets and thrombus formation [165]. Platelets are activated by ADP stimulation of P2Y_1_ and P2Y_12_ receptors and are involved in the regulation of hemostasis and thrombosis. Compound (**84**) turned out to possess the highest antagonist potency against P2Y_1_ with an IC_50_ = 0.21 μM. Authors stated that most of the structure optimization of potent P2Y_1_ receptor antagonist focuses on optimization of the phenoxy group. The most active compound bore 2-tert-butylphenoxy moiety [164].

Sakauchi et al. obtained novel 3,4-dihydro-2*H*-thiochromene-1,1-dioxide derivatives as potent α1D adrenoceptor antagonists [166]. The authors based their syntheses on the fact that many of the drugs currently used in the treatment of benign prostatic hyperplasia, such as Tamsulosin or Silodosin, bore a phenoxyethylamine moiety. Proposed compound (**90**) possessed the highest affinity potency with K_i_ = 0.3 nM for an α1D receptor, as compared to the reference standard with K_i_ = 1.1 nM. The most active compound bore 2,5-difluorophenoxy moiety [166].

## 4. Conclusions

The terminal phenoxy moiety is essential for the activity and pharmacological properties of a large group of drugs. In the case of potential agents for the treatment of neurodegenerative diseases, the phenoxy moiety can mimic amino acid residues, form hydrogen bonds thanks to the ether oxygen atom, which also allows a better fit to the binding pocket, or expand the hydrophobic interactions such as π–π stacking. π-stacking, H–π interactions, and increased selectivity, which translates into lower toxicity, are functions of the phenoxy moiety for agents with anti-cancer activity and BTK inhibitors. In the case of agents with potential antimicrobial activity, the 4-chlorophenoxy group clearly dominates, which is mainly responsible for hydrophobic interactions. 4-substituted-2,6-dimethylphenoxy moiety performs the same function for agents with anti-HIV activity. In the case of antiparasitic agents, the phenoxy moiety also extends the hydrophobic interactions, and its substitution with electron donating groups increases the stability of the new derivatives. Hydrogen bond, π–π stacking, or an increase in metabolic stability are the effects of the phenoxy group in anesthetic agents. Moreover, the presence of two phenoxy groups as a phenoxyphenoxy moiety significantly increases the activity of the compound. The hydrophobic tail of the phenoxy group allows for high selectivity with a simultaneous good membrane permeability, and these are the effects of the phenoxy group on antidiabetic agents. Thus, the terminal phenoxy group has enormous potential for the future of medical chemistry. Therefore, future syntheses of new agents should contain a phenoxy moiety, which could lead to the discovery of new drugs.

## Data Availability

Not applicable.
